# Shall we repeat? Predicting the necessity to repeat blood testing in pediatric emergency departments of laboratory tests previously performed in the community using a machine learning model

**DOI:** 10.1371/journal.pone.0352682

**Published:** 2026-09-21

**Authors:** Adi Shuchami, Teddy Lazebnik, Shai Ashkenazi, Avner Herman Cohen, Yael Reichenberg, Vered Shkalim Zemer

**Affiliations:** 1 Department of Mathematics, Ariel University, Ariel, Israel; 2 Department of Information Systems, University of Haifa, Haifa, Israel; 3 Department of Computing, Jönköping University, Jönköping, Sweden; 4 Adelson School of Medicine, Ariel University, Ariel, Israel; 5 Faculty of Medical and Health Sciences, Tel Aviv University, Tel Aviv, Israel; 6 Dan Petach-Tikva District, Clalit Health Services, Petah Tikva, Central District, Israel; Chittagong Medical College, BANGLADESH

## Abstract

Community‑ordered laboratory blood tests are frequently re-ordered when the patients arrive at the pediatric emergency departments. When only a few hours separate the initial community blood tests from assessment in the emergency department, repeated laboratory testing often does not contribute to clinical management. Additional testing can inconvenience patients, increase out-of-pocket costs for patients and families, add costs to the healthcare system, and delay clinical decision-making. To address this issue, we collected community-issued laboratory tests for complete blood count, electrolytes, and C-reactive protein for children aged three months to 18 years treated in pediatric emergency departments within a nationwide Health Maintenance Organization. These were compared to repeated laboratory tests performed in the pediatric emergency department within 12 hours. Using these data, we first analyzed the frequency of the justified repeated testing according to pre-set criteria. Next, we developed a decision-support machine learning model to predict whether a laboratory test should be repeated, leveraging the patient’s prior community laboratory results and sociodemographic features. For complete blood count, electrolytes, and C-reactive protein, we found that only 13.3%, 16.3%, and 19.0% of the repeated tests, respectively, were deemed justified. Moreover, we show that an out-of-the-box XGboost model can predict the necessity for repeating these laboratory tests with 91.8%, 89.1%, and 79.2% accuracy, respectively. In the absence of false negatives, a condition of high clinical importance, the model maintains accuracies of 73.9%, 67.3%, and 55.4% for the three laboratory categories, respectively. This performance level remains sufficient to substantially reduce unnecessary repeat testing for complete blood count and electrolyte tests, but less so for C-reactive protein. The benefits of the model in clinical practice should be further studied.

## Introduction

Repeat laboratory testing represents a frequent and resource-intensive practice in clinical settings, for example in pediatric emergency departments (PEDs), when children present shortly after laboratory tests have already been performed in the community [1]. From a clinician’s perspective, the motivation for repeated laboratory testing is to obtain the most current diagnostic information, and to facilitate accurate diagnosis and appropriate treatment. However, for pediatric patients presenting to hospital PEDs after a recent community-based blood testing, repeated hospital testing frequently yields results that do not differ significantly from prior community results, thus providing minimal additional diagnostic or therapeutic value. These repeated tests place a burden on healthcare systems, cause discomfort for children, and contribute to parental anxiety [2–5]. However, there is limited research specifically focused on repeated testing among pediatric patients admitted to hospital PEDs, leaving a gap in our understanding of the prevalence, characteristics, and underlying variables of this phenomenon [2,4,5].

Several studies have demonstrated that physicians often have limited awareness of laboratory testing costs and tend to underestimate the extent of test overutilization [6–8]. Among clinical staff, there is a common perception that certain laboratory tests should be frequently repeated, often viewed as necessary to exclude unexpected changes, which may in turn contribute to patients’ management. While the frequency and utility of laboratory testing have been well studied in general hospitals and in the adult population [9–11], these perceptions have not been thoroughly validated in the pediatric population. Fieldston et al [5] addressed this gap, showing that most repeats occur due to missing results and are relatively infrequent. However, repeated testing was found to be redundant in 25%, 35%, and even up to 69% of cases, depending on the specific test, raising the question of when repeated testing is truly justified. Attempts have been suggested to reduce the repeats [12–19]. Repeated laboratory testing during hospitalization is commonly performed as part of routine patient monitoring and represents a different clinical scenario than repeated testing during PED evaluation.

Machine learning (ML) approaches have increasingly been applied in clinical decision-support settings to address similar challenges involving complex, multivariate clinical data [20,21]. For example, Levi et al [22] used an ensemble of the Random Forest [23] and Extreme Gradient Boosting (XGboost) [24] models to predict the risk of lung cancer in both socio-demographic and clinical background information, achieving 71% accuracy for a 5-year prediction. Hasnain et al [25] used an ensemble of several machine learning models to predict post-cystectomy recurrence and survival in bladder cancer patients, showing an accuracy of over 70% for a 5-year prediction.

Laboratory examinations, such as blood tests, are among the most common diagnostic tools used in clinical practice, and their interpretation often requires consideration of multiple factors, including patient history, clinical findings, and prior test results [26]. These data are non-linear, high-dimensional, and highly complex due to the biological and clinical interactions among the different measurements of the blood tests [27–29]. This setting is ideal for a machine learning-based model [30]. Indeed, ML-based models have shown considerable potential for analyzing blood test results, particularly for predicting clinical outcomes and guiding the design and personalization of treatment protocols [31,32]. For instance, Yang et al. [33] developed a Gradient-boosting decision tree-based machine learning model using 27 routine laboratory tests to predict an individual’s SARS-CoV-2 infection status, achieving an area under the receiver operating characteristic (ROC) curve (AUC) of 85%. Meiring et al [34]. demonstrated the ability of ML models to predict intensive care unit mortality on the first day in the unit, with an AUC of 88%, using socio-demographic and blood test information. The time interval between test administration and clinical decision-making is best treated as a decision feature rather than modeled as a time-series variable [35,36].

Regarding the knowledge gap, we aimed to investigate the necessity of repeating blood tests in the PED when the same tests have already been performed in the community a few hours before. This study analyzed paired laboratory tests for children who had community-issued tests followed by a repeated testing in the PED within 12 hours of the community testing. It introduces a machine learning–based decision-support model to assist clinicians in determining whether a repeat test is warranted.

## Materials and methods

The study population included all children aged three months to 18 years who had at least one pair of community and PED laboratory tests performed within 12 hours, during the period from 01/01/2016–31/12/2023 in three central administrative districts in Israel. During each physician’s visit, a diagnosis was recorded according to the International Classification of Diseases, Ninth Revision (ICD-9). Data curation and extraction were performed using structured query language (SQL) queries executed via the MDClone© analytics platform, which accesses electronic medical records from Clalit Healthcare Services (CHS), the primary healthcare provider in the Israeli healthcare system. Both the community- and PED-performed laboratory results for each patient were accessible within the same integrated Clalit Health Services electronic health record system. Patients were not contacted, as the analysis relied solely on pre-identified, existing clinical and administrative records.

The analysis focused on six commonly ordered laboratory tests, grouped into three clinically relevant categories: (1) complete blood count (CBC), which includes white blood cell count, hemoglobin level, and platelet count; (2) serum electrolytes (CHE), including serum sodium and potassium levels; and (3) serum C-reactive protein (CRP). The data collected from the electronic database included socio-demographic information (age, sex, socioeconomic status), results of the laboratory tests performed in the community, and the corresponding results of the laboratory tests performed in the PED. Repeat tests performed after hospital admission were not addressed in the present study. Socioeconomic status (SES) was defined according to the *Socio-Economic Index of Geographical Units* published by the Israeli Central Bureau of Statistics. This index classifies statistical populations into 10 homogeneous clusters (1 = lowest, 10 = highest), based on a composite factor score derived from national demographic, social, and economic administrative data. The median cluster value of the patient’s residential statistical area was assigned [37]. For simplicity, we set scores 1–3 to low, 4–7 to middle, and 8–10 to high SES, aligned with previous studies.

Unfortunately, no established clinical guidelines or previous studies have defined when laboratory tests should be repeated in the PED, on which our pioneering study could have been based. Therefore, to assess whether a repeated test was warranted, we adopted a metric based on a change in measurement status, specifically, when a test result shifted from within the normal range to an abnormal range, indicating a potential change in clinical status and a need for an intervention or treatment. It is important to note that such changes do not always alter the course of treatment and may, in some cases, be redundant from a treatment planning perspective. Nevertheless, we selected this metric because it is well-defined, based solely on laboratory results, and frequently provides timely information that supports clinical decision-making. The normal reference ranges for each blood test were obtained from the Nelson Textbook of Pediatrics, 22^nd^ Edition [38]. In addition, following internal discussions, we used a second criterion to assess the necessity of repeated tests. A test repetition was considered justified if a single test’s value changed by at least 20% in either direction, even while remaining within the normal range. This threshold was chosen to detect a meaningful change in a patient’s clinical trajectory, when formal guidance on repeat testing is unavailable.

The analysis was conducted in three stages. First, we performed descriptive statistical analyses. Second, we carried out a bivariate analysis to examine factors associated with the clinical justification for repeated laboratory tests. Finally, we developed a machine learning model to predict whether a repeated laboratory test would be necessary for a given patient. Descriptive statistics report the distribution of the population’s properties in terms of counts for discrete variables (such as sex), and as means and standard deviations for continuous variables (such as age). Next, all continuous variables were transformed into discrete variables using binning. For the bivariate (contingency-table) analyses, continuous/ordinal variables were discretized into clinically interpretable categories. Age was grouped into pediatric age bands (3 months– < 2 years, 2– < 6 years, 6– < 12 years, and 12–18 years). SES was collapsed from the 10-point national cluster score into low, middle, and high categories. Bivariate analyses were performed separately for each laboratory test category (CBC, CHE, CRP) and for each repeat-necessity definition (normal-to-abnormal change; ≥ 20% absolute change). Associations between categorical predictors (sex, socioeconomic status, residence type, presenting complaint, treating physician, and prior laboratory result categories) and the binary outcome (repeat deemed justified: yes/no) were assessed using Pearson’s chi-square test of independence. When expected cell counts were <5, Fisher’s exact test was used. All tests were two-sided, and statistical significance was defined as p < 0.05 [39].

Finally, an XGBoost model was trained, with a hyperparameter tuning performed using the Tree-based Pipeline Optimization Tool (TPOT), an automated machine learning library [40]. To improve the generalization of the results, we used the k-fold (k = 5) cross-validation method [41] as well as the SAT post-pruning method [42]. Importantly, the population was split into training and validation cohorts, such that the age and sex distributions of both cohorts were statistically similar to ensure that the validation set followed a distribution similar to that of the training set, as required in clinical-related machine learning analysis [43]. This condition was ensured using the Directed Bee Colony Optimization algorithm, following the methodology proposed by Goodman et al. [44]. For the machine learning–based analysis, feature importance was evaluated using the standard permutation feature importance algorithm [45].

We evaluated performance under two clinically-based operating points: (i) an accuracy-optimized scenario, where the probability threshold was selected to maximize overall accuracy, and (ii) a safety-optimized scenario, where the probability threshold was selected to achieve a false negative rate of 0% (i.e., no necessary repeats were missed). Here, a false negative denotes a case in which the model predicts ‘no repeat’ when the repeat was justified by the study definition, and a false positive denotes a case in which the model predicts ‘repeat’ when the repeat was not justified. A sensitivity analysis was also conducted to assess model performance concerning two factors: the time interval (on the hour level) between community-issued laboratory results and the ordering of repeated tests in the PED, and the threshold for defining repeat test necessity based on absolute change. All analyses were conducted using Python, version 3.9.2. No multiplicity adjustment was applied to the bivariate comparisons, which were conducted for descriptive/exploratory characterization; therefore, these p-values should be interpreted cautiously.

### Ethics approval and consent to participate

The study was conducted following the Declaration of Helsinki and approved by the Institutional Review Board of Clalit Health Services, Meir Hospital (approval number: 0099-21-COM). Informed consent was waived by the Institutional Review Board due to the retrospective nature of the study without contacting the patients. No identifying patient data is included (there are no individual details in this manuscript).

## Results

Among the 7,813 patient visits included in the analysis, 6,044 involved repeated CBC tests, 1,941 involved repeated CHE tests, and 2,771 involved repeated CRP tests; some visits included multiple repeated test types. Table 1 summarizes cohort characteristics by laboratory test category. Children with repeated CBC tests were younger on average (mean age 5.08 ± 5.28 years) than those with repeated CHE tests (7.91 ± 5.71 years) or repeated CRP tests (6.33 ± 5.38 years). Sex distribution was approximately balanced across groups (CBC: 2,988 male [49.43%] vs. 2,056 female [50.57%]; CHE: 971 [50.05%] vs. 969 [49.95%]; CRP: 1,452 [52.39%] vs. 1,319 [47.61%]). Socioeconomic status was predominantly low-to-middle (CBC: 1,692 [27.99%] low and 3,125 [51.70%] middle; CHE: 615 [31.68%] low and 903 [46.52%] middle; CRP: 914 [32.98%] low and 1,306 [47.13%] middle), and most participants resided in cities (CBC: 4,219 [69.80%]; CHE: 1,293 [66.61%]; CRP: 1,974 [71.24%]). Pain was the most common presenting complaint (CBC: 2,609 [43.17%]; CHE: 844 [43.48%]; CRP: 970 [35.37%]). Repeat tests were most commonly ordered by pediatricians when they were acting as treating physicians (CBC: 5,108 [84.51%]; CHE: 1,669 [85.99%]; CRP: 2,319 [83.69%]). Repeated tests were classified as justified if results changed from normal to abnormal (CBC: 803 [13.28%]; CHE: 316 [16.28%]; CRP: 527 [19.02%]) or if there was an absolute change of ≥20% (CBC: 442 [7.32%]; CHE: 230 [11.85%]; CRP: 379 [13.68%]). Most repeated tests did not meet our definition of a justified repeat, with only 7.32%–19.02% considered justified depending on the criterion applied.

**Table 1:** Socio-demographic and clinical characteristics of the study’s population (adapted from data reported in Shuchami et al. [46]).

Variable	Repeated CBC	Repeated CHE	Repeated CRP
**Paired testing events, n**	6044	1941	2771
**Age (years), mean (±SD)**	5.08 (5.28)	7.91 (5.71)	6.33 (5.38)
**Sex**			
Male: Female ratio	1.45:1	1.00:1	1.10:1
**SES, n (%)**			
Low	1692 (27.99%)	615 (31.68%)	914 (32.98%)
Middle	3125 (51.70%)	903 (46.52%)	1306 (47.13%)
High	1227 (20.31%)	423 (21.80%)	551 (19.89%)
**Residence type, n (%)**			
City	4219 (69.80%)	1293 (66.61%)	1974 (71.24%)
Village	1458 (24.12%)	495 (25.50%)	638 (23.02%)
Other	367 (6.98%)	152 (7.89%)	159 (5.74%)
**Patient’s complaint, n (%)**			
Fever	2120 (35.07%)	729 (37.56%)	803 (28.98%)
Pain	2609 (43.17%)	844 (43.48%)	970 (35.37%)
Weakness	718 (11.88%)	219 (11.28%)	424 (15.30%)
Other	597 (9.88%)	149 (7.68%)	574 (19.19%)
**Treating physician, n (%)**			
Pediatrician	5108 (84.51%)	1669 (85.99%)	2319 (83.69%)
Family physician	936 (15.49%)	272 (14.01%)	452 (16.31%)
**Repeated test justified according to our definitions, n (%)**			
Normal to abnormal change	803 (13.28%)	316 (16.28%)	527 (19.02%)
Absolute change of ≥20%	442 (7.32%)	230 (11.85%)	379 (13.68%)

Treating physician refers to the emergency room physician who has ordered the repeated testing. CBC – complete blood count test, CHE – electrolyte test, CRP – C-reactive protein.

Based on the study cohort, the association between each sociodemographic and clinical variable and the likelihood of requiring a repeated laboratory test was examined. Table 2 presents the results of the bivariate analysis, including the p-values for each variable concerning the necessity of repeated testing. Age showed nominal (uncorrected) statistical associations across the laboratory test categories under both repetition criteria. Sex was not statistically significant for CHE tests, with p-values of 0.071 and 0.052. As expected, the specific type of laboratory test showed a significant association with the likelihood of repetition. In contrast, other variables such as socioeconomic status, type of residence, primary complaint, and referring physician showed no significant association with the necessity of repeating laboratory testing under either criterion.

**Table 2:** Bivariate analysis between the socio-demographic and clinical properties of individuals and the need to repeat laboratory tests.

	Normal to abnormal change			Absolute change of 20%		
	CBC	CHE	CRP	CBC	CHE	CRP
Age	0.008*	0.045*	0.038*	0.016*	0.061	0.038*
Sex	0.110	0.071	0.080	0.102	0.052	0.066
SES	0.228	0.271	0.236	0.148	0.101	0.127
Residence type	0.408	0.184	0.279	0.343	0.391	0.272
Patient complaint	0.084	0.092	0.102	0.070	0.086	0.109
Treating physician	0.683	0.497	0.504	0.309	0.461	0.288
CBC results	0.027*	0.168	0.205	0.018*	0.129	0.160
CHE results	0.182	0.034*	0.184	0.150	0.025*	0.193
CRP results	0.221	0.174	0.023*	0.152	0.167	0.013*

* p < 0.05, p-values were computed using Pearson’s chi-square test. CBC – complete blood count test, CHE – electrolyte test, CRP – C-reactive protein. *Treating physician refers to the emergency room physician (pediatrician or family physician) who has ordered the repeated testing.*

The performance of the machine learning model was evaluated in terms of accuracy and false negative rates across both repetition criteria and laboratory test categories. To reflect different clinical priorities, results are reported under two complementary scenarios (Table 3). Under the accuracy-optimized scenario, the model achieved strong results for CBC tests, reaching approximately 92% accuracy for the normal-to-abnormal change criterion and 90% for the ≥ 20% absolute change criterion, with similar performance for CHE tests (89% and 90%, respectively). In contrast, CRP tests showed a noticeable decline in performance, with accuracy dropping to 79% and 80%, respectively. When the false negative rate was constrained to zero, the average accuracy across both repetition criteria decreased to 75% for CBC, 68% for CHE, and 56% for CRP. Due to the binary nature of the prediction task, comparison with a 50% random-guess baseline showed that the model provided a substantial predictive gain for CBC (around 25 percentage points) and CHE (around 18 points), but a relatively smaller gain for CRP (around 6 points). Moreover, because the outcome is highly imbalanced (only ~7–19% of repeats are classified as justified depending on criterion), a majority-class baseline yields high nominal accuracy but would miss all necessary repeats. Therefore, we focus on performance under clinically meaningful operating points, including a zero–false-negative (FNR = 0) scenario to prioritize safety.

**Table 3:** Machine learning model’s performance.

Decision threshold scenario	Repeating metric	Laboratory test*	Accuracy	False negative rate
Accuracy-optimized (FN allowed)	Normal to abnormal change	CBC	91.8%	10.2%
		CHE	89.1%	16.3%
		CRP	79.2%	13.6%
	≥20% absolute change	CBC	92.6%	8.0%
		CHE	90.2%	14.9%
		CRP	80.5%	11.1%
Safety-optimized (FN = 0%)	Normal to abnormal change	CBC	73.9%	0%
		CHE	67.3%	0%
		CRP	55.4%	0%
	≥20% absolute change	CBC	75.6%	0%
		CHE	68.5%	0%
		CRP	57.0%	0%

CBC – complete blood count test, CHE – electrolyte test, CRP – C-reactive protein.

To contextualize clinical relevance, we estimated the number of repeated tests that could be avoided if the model were used as a decision support. Under the safety-preserving operating point (FNR = 0), the model would recommend not repeating approximately 3,664/6,044 CBC (60.6%), 990/1,941 CHE (51.0%), and 1,008/2,771 CRP (36.4%) tests under the normal-to-abnormal criterion. Under the ≥ 20% absolute-change criterion, the corresponding estimates were 4,127/6,044 CBC (68.3%), 1,100/1,941 CHE (56.7%), and 1,200/2,771 CRP (43.3%).

**Fig 1:**
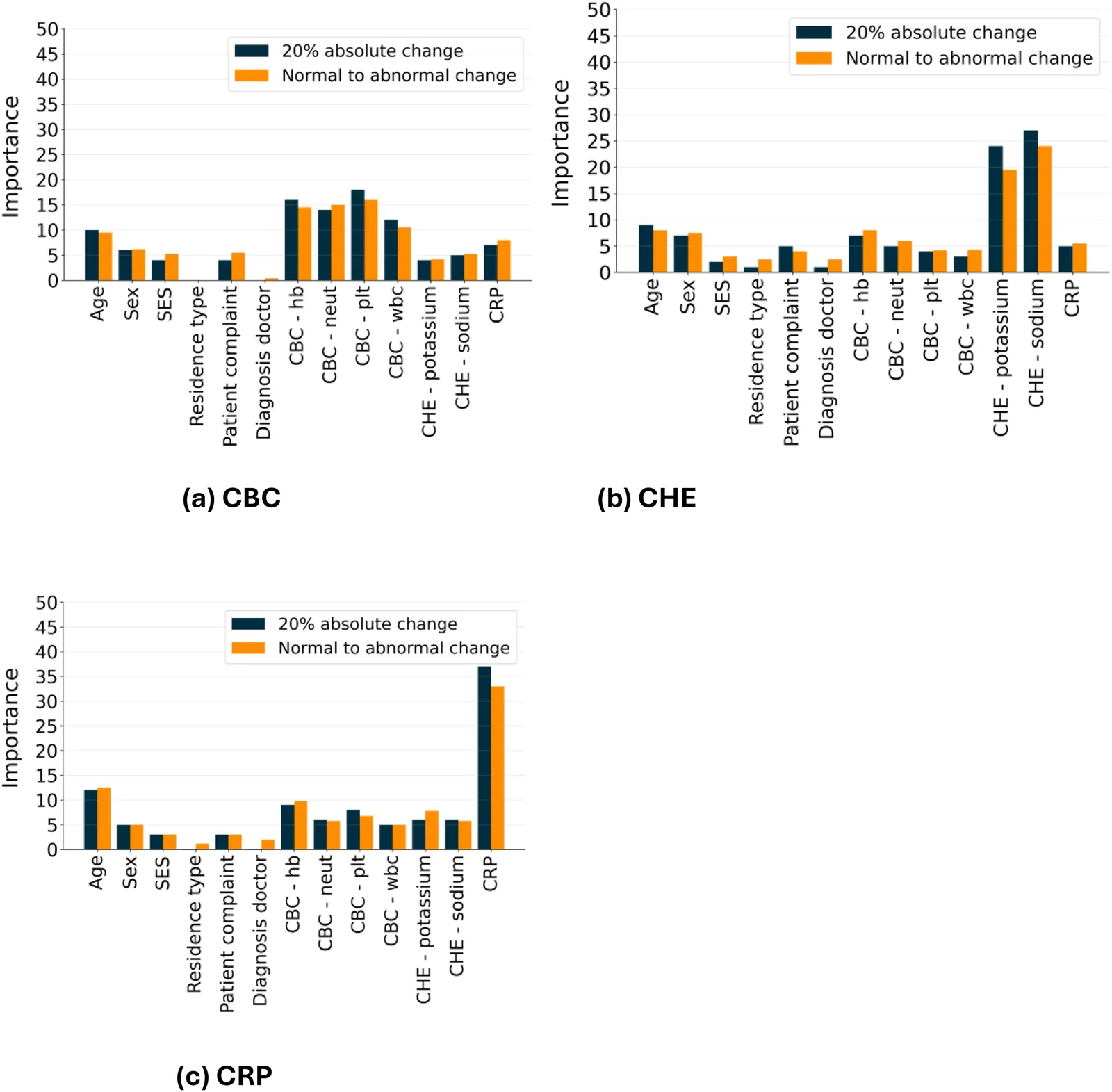
Feature Importance of the Machine Learning Models by Laboratory Test Category.

Fig 1 outlines the feature importance distribution for both the ≥ 20% absolute change and the normal to abnormal switch. Across the three laboratory test types, the feature importance distributions were found to be similar under both repetition criteria, suggesting that the models remained relatively stable across metrics. Age contributed 10% to the model’s predictions, followed by sex, which contributed 6%. In contrast, socioeconomic status and process-related features made only a minor contribution. When examining prior laboratory test results from the community, the most influential feature for each model was the previous result of the same test type, although other test types also contributed meaningfully to the predictions.

**Fig 2:**
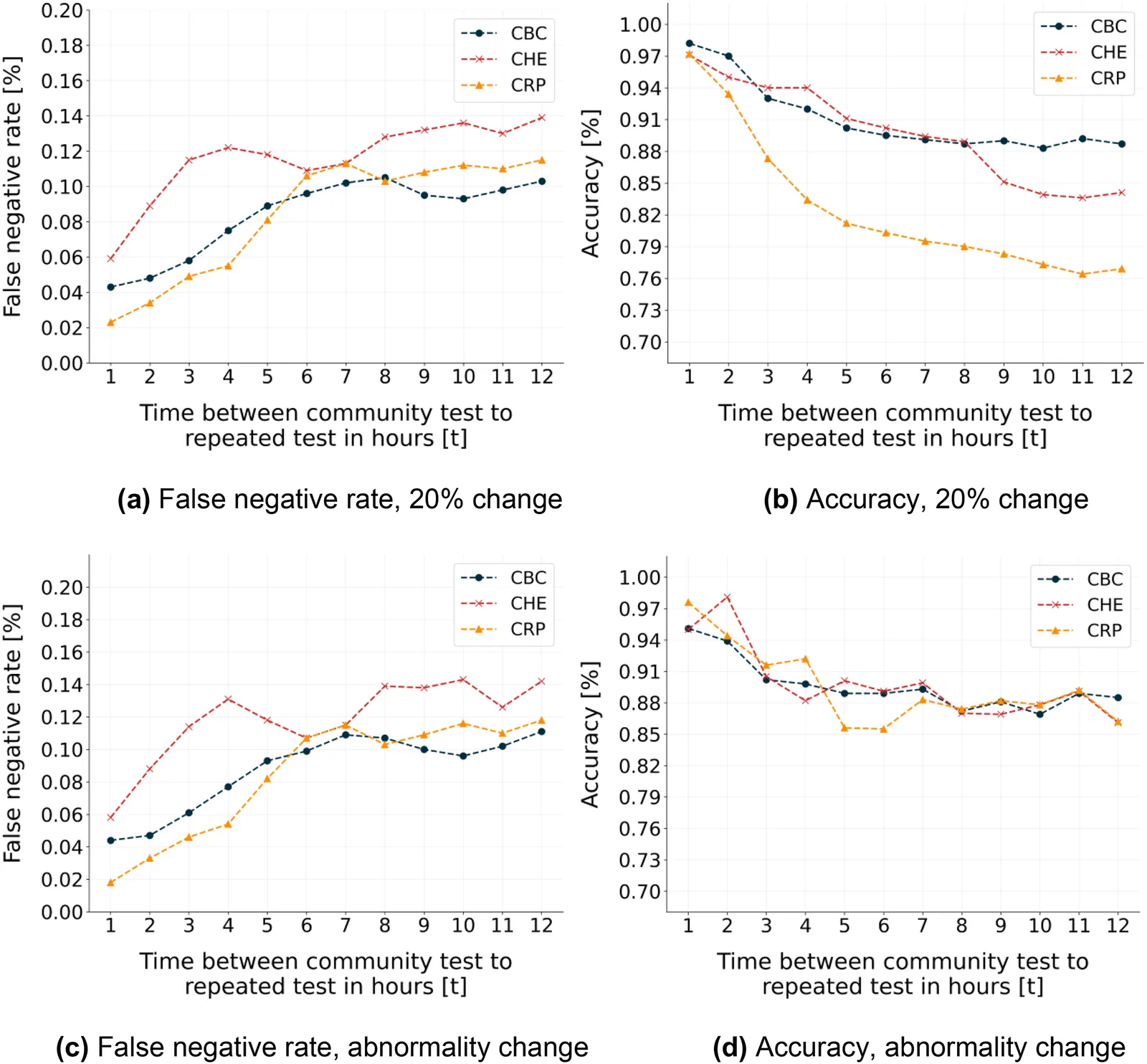
Model performance in predicting the need for repeated testing, measured by false negative rate and accuracy, for both the ≥ 20% absolute change criterion and the normal-to-abnormal change criterion.

Fig 2 presents the performance of the machine learning model, showing both false negative rate and accuracy for the ≥ 20% absolute change and the normal-to-abnormal change definitions. Performance is displayed as a function of the time interval between the community-issued test results and the time at which a repeated test was ordered in the hospital, for each laboratory test type. As the time interval between the initial laboratory test and the subsequent decision to repeat it increases (i.e., right on the x-axis), the models tend to produce higher false negative rates and lower accuracy. However, this pattern is not strictly monotonic and differs slightly between the two repetition definitions.

**Fig 3:**
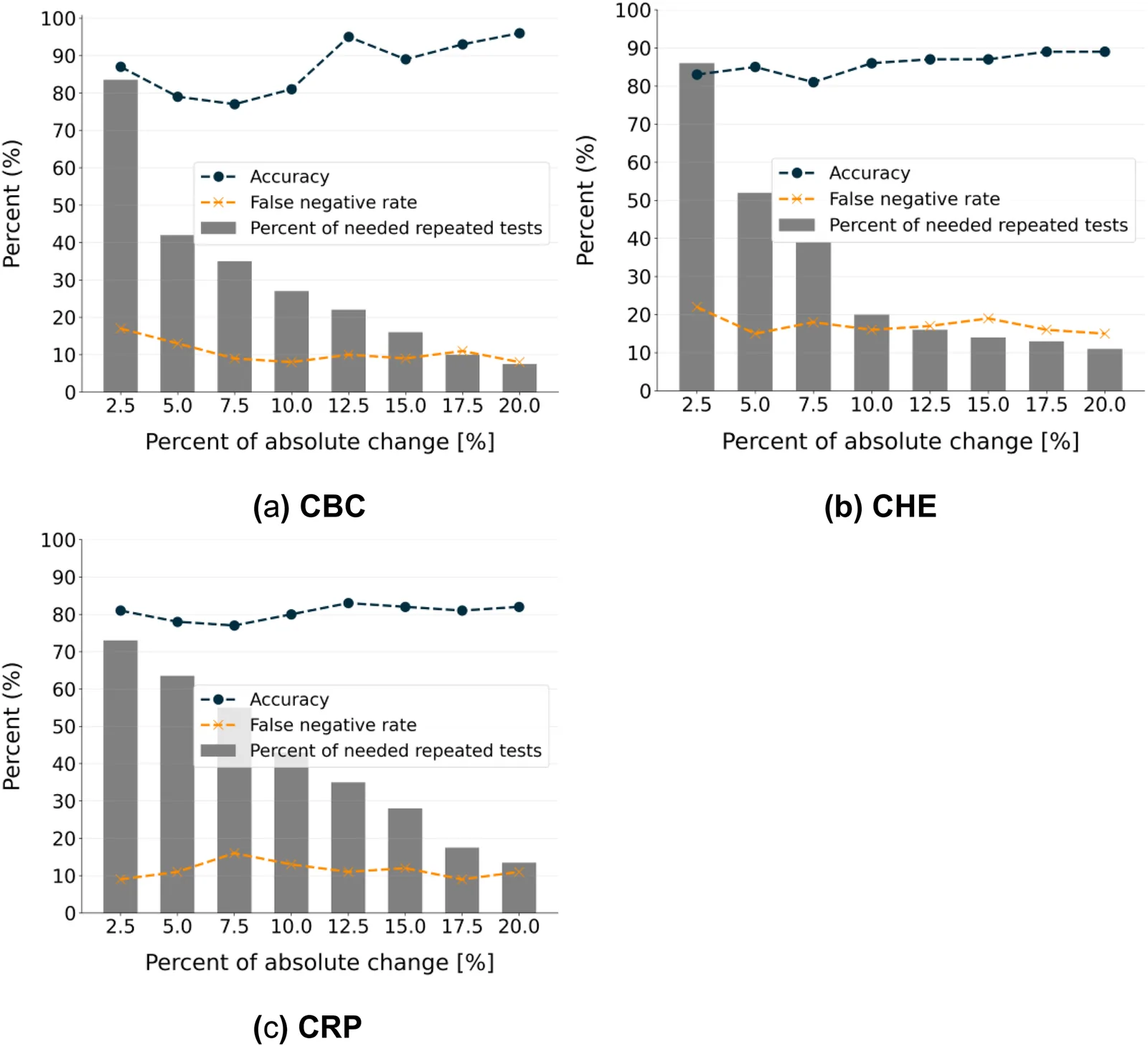
Proportion of justified repeated testing and model performance across absolute change thresholds by laboratory test category.

Fig 3 illustrates the relationship between the proportion of the justified repeated testing according to our pre-set definition and the model’s performance metrics, including accuracy and false negative rate, as a function of varying thresholds of absolute change. For each laboratory test category, the Figure uses bars to show the proportion of tests classified as necessary and lines to show the corresponding model performance (accuracy and false negative rate) at each threshold. The analysis is presented separately for each of the three laboratory test categories. The bars represent the percentage of repeated tests classified as justified. The blue dotted line shows the accuracy of the machine learning model, while the orange solid line depicts the model’s false negative rate. Notably, the proportion of justified repeated tests decreases steadily as the threshold for absolute change increases across all three laboratory test categories. Furthermore, the model’s accuracy improves as the absolute change threshold increases. This improvement stems from growing class imbalance. The stable false negative rate suggests that cases presenting with marked severity supply clear diagnostic signals, thereby increasing model accuracy.

## Discussion and conclusions

This study investigated the prevalence and characteristics of laboratory tests that were initially performed in the community and subsequently repeated in the pediatric emergency department. Repeated testing can lead to increased healthcare costs, increased patient out of pocket costs, increased patient discomfort, unnecessary delays in clinical decision-making and may contribute to higher hospital admission rates [4,5,17,47,48]. To address these challenges, a machine learning–based predictive model was developed and systematically evaluated, to assist in determining whether repeated testing in the emergency setting is necessary.

As repeated laboratory testing is commonly used in clinical practice [2,8,11,49,50]. Multiple interventions designed to reduce unnecessary laboratory testing have been reported in the medical literature. These interventions include physician education, clinical criteria, audit and feedback, electronic health record notifications, cost information displays, financial incentives, and administrative guidelines that restrict certain types of test ordering, among others [12–15,51,52]. However, the availability and applicability of these interventions in pediatrics are limited [16,17,48]. There are other unique variables considered when repeating tests in the pediatric population, beyond mere cost, which is usually the primary driver in the adult population. These variables include limited anamnesis and physical examination findings, parental anxiety and reassurance, limited/difficult child’s vascular access, and children’s emotional trauma as a result of repeated tests [18,19].

The application of machine learning in medicine has rapidly evolved, offering new possibilities for improving patient care, optimizing clinical workflows, and enhancing diagnostic accuracy [20,27,29]. Machine learning-based models have shown promise in analyzing complex datasets, identifying patterns that may not be readily apparent to human clinicians, and making predictions that can inform clinical decision-making [21,22,25]. Previous studies on machine learning models for predicting blood test outcomes have consistently demonstrated the superior predictive performance of XGBoost compared to other methods [35].

Our results indicate that the proportion of repeated laboratory tests considered appropriate according to our pre-set definitions, is relatively low, approximately 10%, 14.5%, and 16.5% for CBC, CHE, and CRP tests, respectively, averaged across both the normal to abnormal change and ≥20% absolute change criteria. These findings are consistent with those reported in previous studies. In the pediatric setting, Fieldston et al. [5] found that repeated testing was redundant in 25% to 69% of cases depending on the specific test, and Coon et al. [4] identified repeat testing as a key component of pediatric overuse more broadly. Similar patterns have been documented in adults: Kandalam et al. [49] reported that approximately 36% of repeated electrolyte panels within 24 hours in Canadian hospitals had previously normal results, and Murphy et al. [51] found that 24% of CBCs and 25% of electrolyte panels among adult inpatients were ordered inappropriately using validated criteria. Notably, Gornik et al. [47] demonstrated that unnecessary repetitions of CRP and leukocyte counts in an ED observation unit were associated with higher hospital admission rates, suggesting that repeated testing may paradoxically drive clinical decisions. Intervention studies further support the need for decision support tools: Anderson et al. [52] showed that implementing a CDS alert reduced inappropriate blood chemistry orders from 28.64% to 15.69% (p < 0.001), consistent with broader evidence that structured interventions can meaningfully reduce unnecessary testing [12–15,51]. However, as Fig 3 demonstrates, these proportions vary greatly depending on the threshold used for absolute change, ranging from as high as 83.4% at a 2.5% threshold to as low as 7.3% at a 20% threshold. This variability highlights the limitation of using fixed numerical thresholds alone, such as values inside or outside normal ranges, to determine test necessity. Instead, it points to the importance of defining test repetition criteria based on their actual impact on clinical outcomes, in our path towards a personalized approach [31]. In Israel, performing these tests costs the healthcare system between 3.13$ and 41.33$. For our cohort, patients incurred approximately $8,000 in costs for apparent unjustified repeated testing according to our definitions. Although this amount may initially seem modest in clinical terms, the frequent use of these tests causes the total expenses to accumulate rapidly, resulting in significant and avoidable costs [48,53]. This estimate excludes healthcare professionals’ time and other operational costs, which are difficult to quantify and highly variable, yet contribute to the overall expense of unnecessary laboratory testing and the burden on the healthcare system [19]. Additionally, excessive testing may reduce patient and family satisfaction with healthcare providers, potentially resulting in adverse clinical and financial outcomes [48,53].

### Strengths and limitations

A key strength of this study is its real-world, population-based design. To our knowledge, our previous report [46] and the present complementary study are the first comprehensive investigation of repeated blood tests in pediatric patients across community and emergency department settings to utilize a machine learning model for analysis. However, multiple limitations warrant consideration. First, the analysis was limited to six commonly ordered blood tests (grouped into three clinically-relevant categories: CBC, CHE, and CRP), which may not capture repeat testing patterns for other laboratory assays. Second, the model did not include patients’ detailed clinical records, data accessible to treating physicians that offer crucial insight into the patient’s condition and the decision-making process for ordering repeated tests in the emergency department. Including clinical data could enhance the model’s predictive accuracy. Changes in clinical parameters of the patients could have justified the repeated laboratory testing by the physician. Also, in this study, repeat testing was defined as any test performed within 12 hours of the initial test and that ordered during the PED visit. This strict definition focuses on a specific subset of repeat tests, rather than encompassing all repeated testing that may occur throughout a patient’s entire hospital stay or over a longer period of interaction with the healthcare system. As such, future studies are planned to focus on different time windows, during treatment and other settings. Finally, the study did not assess the impact of repeat testing on patients’ clinical outcomes, limiting our ability to draw definitive conclusions about the clinical significance of repeated testing within the overall care process.

### Conclusions and future plans

In our cohort, we applied pre-defined objective criteria, either a transition from a normal to an abnormal value or an absolute change ≥20% to justify repeated testing of common blood examinations (CBC, CHE, and CRP). The proportion of repeated laboratory tests classified as justified according to our criteria was low, ranging from 7.32% to 19.02%, depending on the test type and the applied criterion, indicating that over 80% of repeat tests did not meet our definition of a justified repeat. The implementation of the machine learning-based predictive model improved the identification of justified repeated tests to 75.6%, 68.5%, and 57.0% for the CBC, CHE, and CRP, respectively, without missing any cases requiring repetition. This advancement has the potential to significantly reduce healthcare burden, costs and to shorten treatment delays while maintaining high standards of patient care. The tool is intended to assist the pediatrician, remembering that the clinical judgment and the patient’s condition remain essential in the decision-making. Future research should focus on detailed patient-level analyses to refine this approach and assist healthcare providers in making efficient data-driven decisions and elucidate the benefits of the suggested model in clinical practice.

## Abbrevations:

AUC: Area Under the Curve
CBC: Complete Blood Count
CDS: Clinical Decision Support
CHE: Serum Electrolytes
CHS: Clalit Health Services
CRP: C-Reactive Protein
DT: Decision Tree
FNR: False Negative Rate
ML: Machine Learning
PED: Pediatric Emergency Department
ROC: Receiver Operating Characteristic
SARS-CoV-2: Severe Acute Respiratory Syndrome Coronavirus 2
SES: Socio-Economic Status
SQL: Structured Query Language
TPOT: Tree-based Pipeline Optimization Tool
XGBoost: Extreme Gradient Boosting
